# Low-Level Domoic Acid Exposure Induces Age-like Cardiomyopathy in Young Adult and Aged Mice

**DOI:** 10.3390/md24060210

**Published:** 2026-06-13

**Authors:** Sophia Liu, Alicia Hendrix, James MacDonald, Theo Bammler, Kathi A. Lefebvre, David J. Marcinek

**Affiliations:** 1 Department of Radiology, University of Washington Medical School, Seattle, WA 98109, USA; sophia21@uw.edu (S.L.); dmarc@uw.edu (D.J.M.); 2 Department of Laboratory Medicine and Pathology, University of Washington Medical School, Seattle, WA 98109, USA; 3 Pacific Islands Fish and Wildlife Office, U.S. Fish and Wildlife Service, Honolulu, HI 96850, USA; alicia_hendrix@fws.gov; 4 Department of Environmental and Occupational Health, University of Washington, Seattle, WA 98109, USA; jmacdon@uw.edu (J.M.); tbammler@uw.edu (T.B.); 5 Environmental Chemistry Program, Environmental and Fisheries Science Division, Northwest Fisheries Science Center, National Marine Fisheries Service, National Oceanic and Atmospheric Administration, Seattle, WA 98112, USA

**Keywords:** harmful algal bloom (HAB), subacute exposure, chronic, excitotoxicity, seafood toxins

## Abstract

Domoic acid (DA) is a well-known seafood toxin produced by some species of marine phytoplankton in the genus *Pseudo-nitzschia* during harmful algal blooms (HABs). Acute toxic exposures induce overt clinical signs of neuroexcitotoxicity, such as seizures in mammals due to overstimulation of glutamate receptors in the central nervous system (CNS). Acute DA excitotoxicity via the CNS has been well-studied in both field poisoning events and laboratory exposure studies with rodent models, but little is known about the impacts of low-level DA exposures below those that cause outward signs of neurotoxicity; the impacts on other potential target organs, including the heart; or age-related sensitivities. Here, low-level DA exposures in young adult (9 mo) and old (24 mo) mice were conducted over multiple weeks. Mortality, cardiac function, frailty, and protein expression were quantified to assess age-related DA sensitivity and potential impacts on heart function. Echocardiography and proteome data confirm that chronic low-level DA exposure causes irreversible functional cardiomyopathy and protein remodeling in young adult mice that mimics natural cardiac aging. In addition, old mice exhibit higher mortality and frailty than young adult mice with the same low-level DA exposures. These results provide critical information for assessing potential health risks to humans who regularly consume seafood with low levels of DA.

## 1. Introduction

Domoic acid (DA) is a neuroexcitotoxic glutamate agonist that is naturally produced by some diatoms in the genus *Pseudo-nitzschia* during harmful algal bloom (HAB) events [1,2]. During HABs, filter-feeding organisms such as clams and planktivorous fish can accumulate DA and transfer the toxin through food webs to higher trophic level consumers [3,4]. Consumption of DA-contaminated seafood can profoundly impact marine wildlife and cause the human illness known as Amnesic Shellfish Poisoning (ASP), which is characterized by gastrointestinal distress, cognitive impairment, confusion, seizures, and, in the most severe cases, death [4,5]. As the global population whose diet depends on marine systems nears three billion [6], it is critical to develop a more thorough understanding of this seafood-borne toxin and its potential threats to public health.

Many recent studies suggest that the presence of DA will become more consistent and persistent in food webs across a broader geographic range in years to come [7,8,9,10,11], due to increases in HAB occurrence and severity as ocean conditions shift with climate change [11,12]. While acute high-level DA exposure has been the focus of most research, chronic exposures to low levels of DA may become increasingly more common as a result of increased prevalence of DA in the food web [13]. Repeated consumption of DA at levels below those that cause overt neurotoxic symptoms has already been shown to impact human health: reports from the Communities Advancing the Studies of Tribal Nations Across the Lifespan (CoASTAL) cohort describe associations between regular consumption of razor clams with low DA concentrations and poorer performance on certain memory tests [14,15,16]. While some laboratory rodent and nonhuman primate models have followed up on this work, reporting changes in activity levels, memory function, motor coordination, and aggression depending on exposure regimen [17,18,19,20], the literature in the area is still quite limited. Interestingly, some subtle effects of repeated DA exposure may be reversible; the memory impairments and activity changes in mice exposed to subconvulsive DA doses for 25 weeks were reversed after a 9-week recovery period of no exposure [20].

In addition to assessing the relationship between chronic low-level DA exposure and neurological function, it is also necessary to assess potential toxicity to peripheral organ systems. Organs as diverse as the heart, kidney, liver, lung, reproductive organs, and spleen express the glutamate receptors that DA binds to and overstimulates, and could therefore be impacted by exposure to DA [21,22]. Safety regulations for allowable maximum DA concentrations in seafood used for human consumption of <20 μg/g shellfish have been effective in reducing ASP cases [23]. However, these regulations do not address long-term low-level DA exposure from consuming seafood with DA levels below regulatory limits [10,24]. As the potential for human exposure to DA increases, a better understanding of the effects of chronic exposure to levels below those eliciting acute excitotoxicity and how these effects interact with other factors, such as underlying disease and age, will become more important in managing potential health impacts [4,25].

The present study reports results from two experiments testing the effects of chronic low-level exposure to DA on cardiac function in young adult and old female mice. Both long (13 weeks)- and short (three weeks)-term DA exposures to doses below those that cause outward signs of neurobehavioral excitotoxicity were shown to cause irreversible deficits in diastolic function in young adult and aged mice. Additionally, exposure in young mice elicited changes in the cardiac proteome similar to those observed with natural aging.

## 2. Results

### 2.1. Long-Term Low-Level DA Exposure Experiment (13 Weeks)

#### Long-Term DA Exposure Impairs Cardiac Function

**Figure 1:**
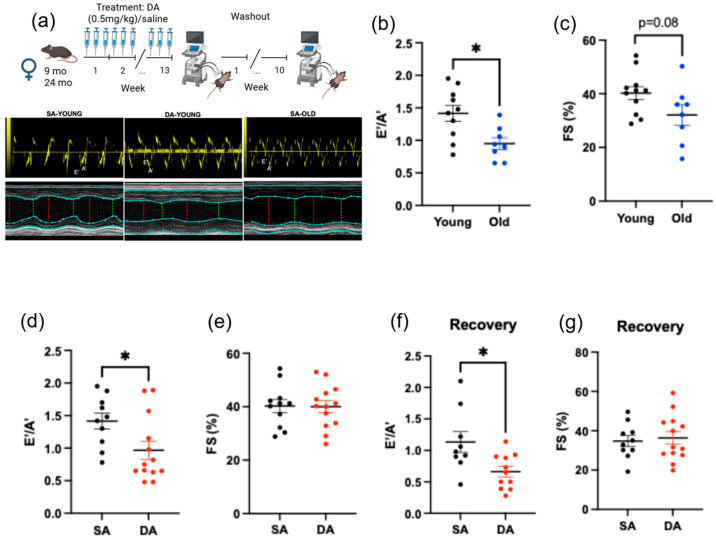
Reduced cardiac function after 13-week domoic acid (DA) exposure. (**a**) Study schematic of long-term low-level DA exposure and recovery period and example images from echocardiography used to calculate diastolic function (E’/A’, (**top row**)) or systolic function (% fractional shortening, (**bottom row**)). (**b**) Aging effect on diastolic function represents early to late diastole ratio (E’/A’) in young and old mice (no exposure). Diastolic function of blood flow across the mitral valve in early diastole (Ea/E’) to the flow in late diastole (Aa/A’). (**c**) Aging effect on systolic function represented by fractional shortening (FS%) in young and old mice (no exposure). (**d**,**e**) E’/A’ and FS% after 13-week DA exposure in young mice (SA = saline-treated; DA = domoic acid-treated). (**f**,**g**) E’/A’ and FS% after 10-week recovery period (no exposure) in young mice. * *p* < 0.05.

Young adult (9 mo) and aged female mice (24 mo) were exposed to sub-convulsive intraperitoneal (IP) injection doses of 0.5 mg/kg bw DA or saline (media only) three days a week for 13 weeks. In vivo echocardiography was performed to assess cardiac function at the end of week 13, then repeated following a ten-week recovery period of no exposure (Figure 1a). Only 31% of DA-exposed aged mice survived the exposure period (Table 1) compared to a 82% survival rate in the saline group. Due to the low survival rate, reliable echocardiography data for the 13-week and recovery period for the aged mice were not available (Table 1). Quantifications of systolic function via fractional shortening (FS) using the M-mode short axis and diastolic function (E’/A’) via the ratio of blood flow across the mitral valve in early diastole (E’) to the flow in late diastole (A’) were used to assess cardiac function (Figure 1a). E’/A’ was significantly reduced, and FS tended to be reduced with age in the saline-treated mice (Figure 1b,c). In the young adult mice, E’/A’ was significantly reduced in DA-exposed mice (Figure 1d), but there was no effect of DA treatment on FS (Figure 1e). Reduced E’/A’ in DA-treated young mice mirrored the effect of age on E’/A’ (Figure 1b,d) and persisted after the ten-week recovery period (Figure 1f). There was no effect of 13-week DA exposure on FS in young mice after the ten-week recovery period (Figure 1g).

### 2.2. Short-Term Low-Level DA Exposure Experiment (Three Weeks)

#### 2.2.1. Short-Term DA Exposure Impairs General Health Status

**Figure 2:**
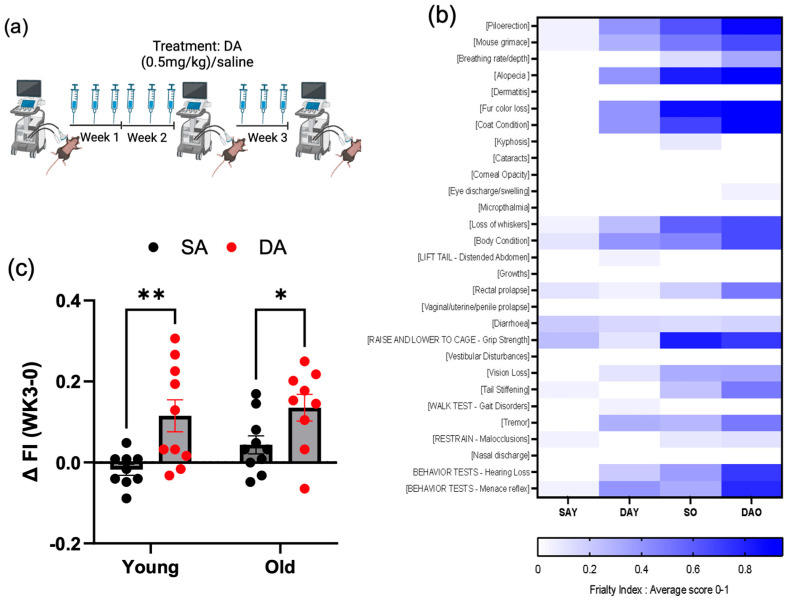
Frailty increases after three-week domoic acid (DA) exposure in both young adult and aged mice. (**a**) Study schematic of short-term DA exposure (three weeks). (**b**) Heat map showing frailty index (FI) parameters and severity after three weeks of DA or saline (SA) injections in young (Y) and old (O) mice. (**c**) Endpoint frailty index in young and old mice after three weeks of exposure (IP injections of saline (SA) or DA three times per week). * *p* < 0.05, ** *p* < 0.01.

**Figure A1:**
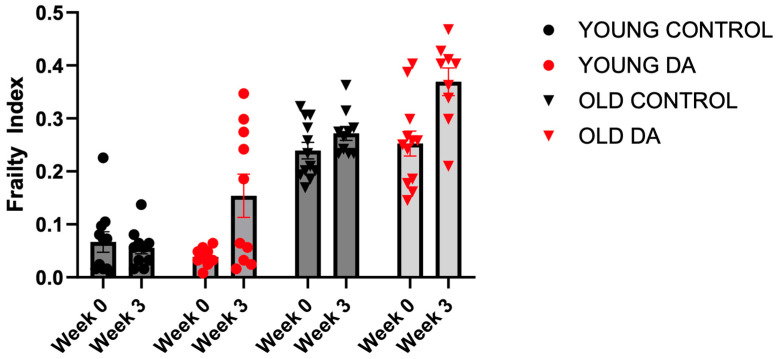
Frailty index in young adult and old mice at baseline and after three weeks of treatment (domoic acid (DA) or Saline (S) controls). Frailty index was assessed in young control (black circles), young DA-treated (red circles), old control (black triangles), and old DA-treated (red triangles) mice at baseline (week 0) and after three weeks of injection. Each point represents an individual animal, and bars indicate mean ± SEM. Aged mice exhibit higher frailty compared to young mice, with changes over the 3-week intervention period reflecting treatment- and age-related effects.

In the short-term DA exposure experiment, frailty index (FI) scores were used as a measure of general health status at baseline and after three weeks of exposure (Figure 2a). As expected, FI scores were higher in the aged mice compared to young mice (Figure 2b and Appendix A Figure A1). Three weeks of DA exposure increased FI scores in both young and old mice, while there was no change in the saline-treated groups (Figure 2c). DA exposure affected a diverse range of frailty index components, including physical appearance (e.g., coat and body condition) and physiological (e.g., tremor and piloerection), and behavioral (e.g., menace reflex and tail stiffening) characteristics in both young and aged mice (Figure 2b).

#### 2.2.2. Short-Term DA Exposure Impairs Cardiac Function

**Figure 3:**
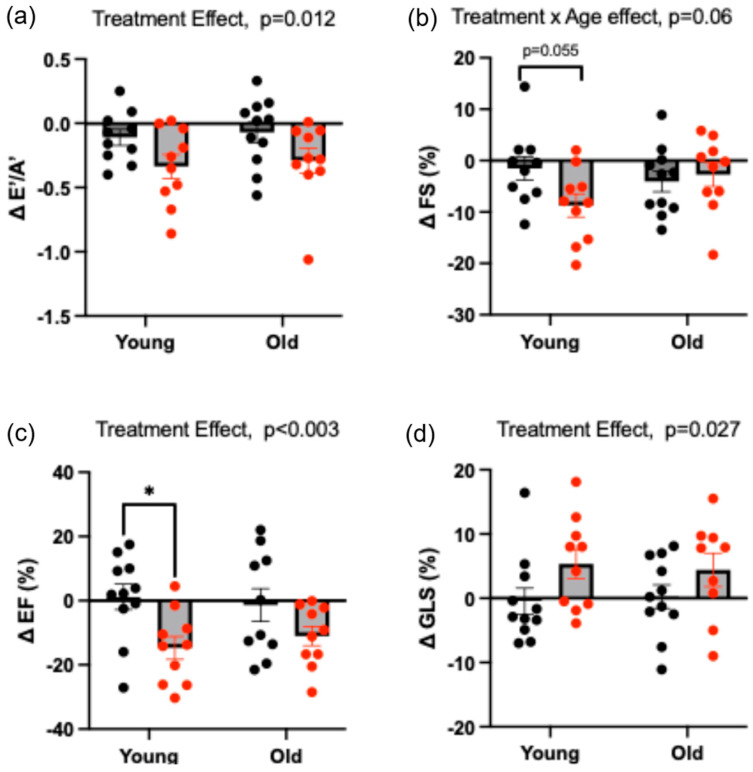
Cardiac function changes after three-week domoic acid (DA) exposure in young adult and old mice. (**a**) Early to late diastole ratio (E’/A’), (**b**) % fractional shortening (FS), (**c**) % ejection fraction (EF), and (**d**) global longitudinal strain (% GLS) changes (three-week minus baseline values). Red dots = domoic acid treatment. Black dots = saline treatment. Treatment and interaction effects tested by two-way ANOVA or mixed model. * *p* < 0.05.

**Figure A2:**
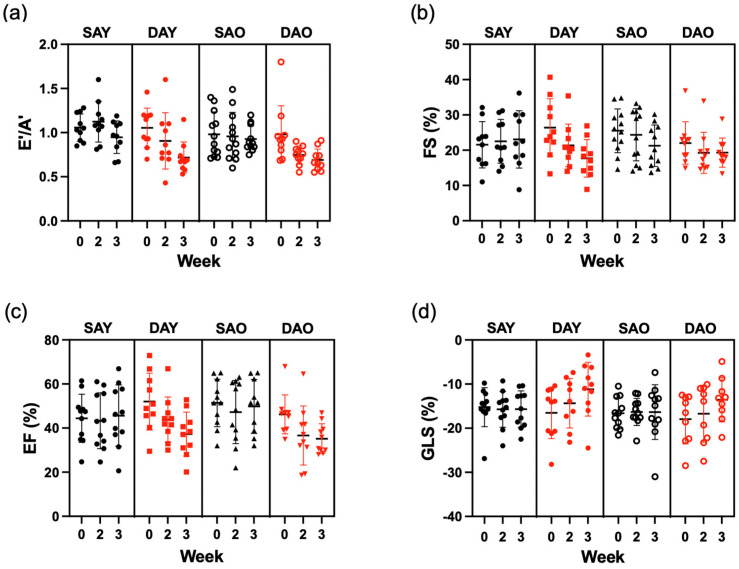
Changes in cardiac diastolic (E’/A’) function and systolic function in young and aged mice following three weeks of treatment. (**a**) E’/A’ ratio, (**b**) fractional shortening (FS, %), (**c**) ejection fraction (EF, %), and (**d**) global longitudinal strain (GLS, %) measured at baseline (week 0), week 2, and week 3 in young (Y) and aged (O) mice. Data are presented for saline-treated young (SAY), domoic acid (DA)-treated young (DAY), saline-treated aged (SAO), and DA-treated aged (DAO) groups. Each point represents an individual animal; horizontal lines indicate mean ± SD. These data illustrate longitudinal changes in diastolic and systolic cardiac function and age-related differences in response to treatment over the three-week intervention period. Black symbols = saline-treated. Red symbols = DA-treated.

In the short-term DA exposure experiment, in vivo cardiac function was assessed via echocardiography at baseline (pre-treatment) and after two (Appendix A Figure A2) and three weeks of three times per week DA or saline IP injections (Figure 2a). In addition to E’/A’ and FS quantifications as performed in the long-term experiment, global longitudinal strain (GLS) analyses were performed as a more sensitive measure of cardiac systolic function and used to calculate ejection fraction (EF). There was no effect of age at baseline for any of the cardiac function parameters (E’/A’, FS%, EF%, and %GLS) in this cohort of mice (Appendix A Figure A2). To test the effect of DA exposure, changes in function from baseline at three weeks were compared (Figure 3). There was a significant treatment effect of DA for diastolic (E’/A’) (Figure 3a), systolic function (EF) (Figure 3c), and global longitudinal strain (GLS) (Figure 3d). More negative values for GLS indicate greater deformation of the heart during systole and therefore greater systolic function, while more positive GLS values indicate less deformation during systole and lower systolic function (Figure 3d). There was a non-significant trend toward a treatment by age interaction for FS driven primarily by the effect of DA on FS in young mice (Figure 3b). Absolute values for baseline and two- and three-week measurements are presented in Appendix A, Figure A2.

#### 2.2.3. Short-Term DA Exposure Alters Cardiac Proteome Similar to Aging

**Figure 4:**
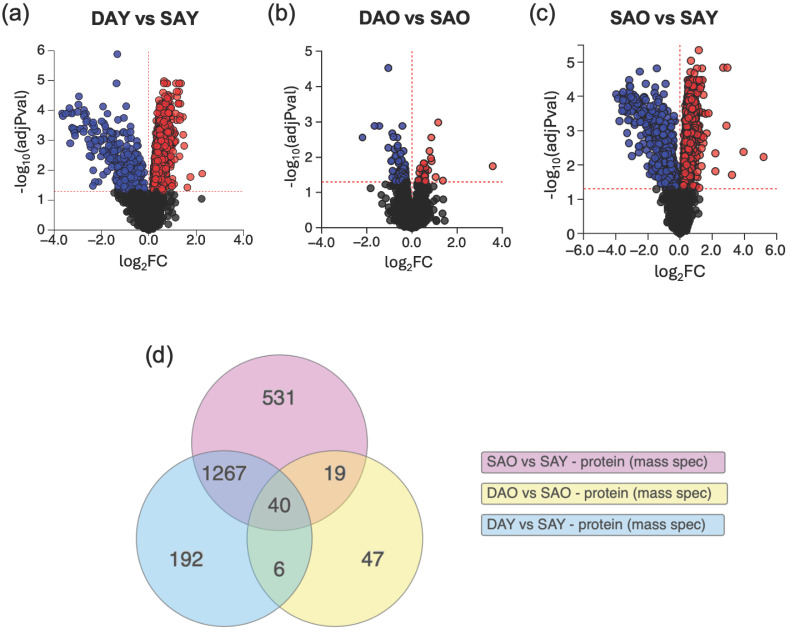
Proteomics after three-week domoic acid (DA) exposure in young adult and old mice. (**a**–**c**) Volcano plots showing differential protein expression in DA-treated young (**a**), DA-treated aged (**b**), and (**c**) aging only. Blue represents downregulation, red represents upregulation, and black indicates no difference. (**d**) Venn diagram depicting overlap of significantly differentiated proteins among saline old (SAO) vs. saline young (SAY), DA old (DAO) vs. saline old (SAO), and DA young (DAY) vs. saline young (SAY).

To test how DA exposure differently remodels the cardiac proteome in young adult and aged hearts, mass spectrometry for proteome quantification was performed with both young adult (Y) and old (O) mice heart extracts. A total of 1505 and 112 differentially expressed proteins were identified in the DA vs. saline (SA) treated young (DAY vs. SAY; Figure 4a) and old (DAO vs. SAO; Figure 4b) mouse hearts, respectively. Comparing young adult to aged saline-treated hearts identified 1857 differentially expressed proteins (SAO vs. SAY; Figure 4c). There was substantial overlap between the effects of DA in the young (blue circle) and the effects of age in the saline-treated group (pink circle), with 1307 proteins in common (Figure 4d; pink/blue intersection). Effects of DA in the aged (Figure 4d; yellow circle) and effects of aging alone (Figure 4d; pink circle) only shared 59 proteins, and only 40 were different in all three comparisons (Figure 4d). Pathway analysis was performed to assess the common pathways that were shared and unique between the groups. There were 23 pathways that were significantly altered by either DA or age or both (Table 2). Fifteen pathways were shared between aging and DA treatment in the young (Table 2, bold font). Consistent with the relatively few differentially expressed proteins, there were no pathways significantly altered by DA treatment in the aged mice (Table 2; Old-DA vs. Old-S column).

## 3. Discussion

Our results indicate that repeated low-level exposure to DA increased biological age and significant detrimental health effects in both young adult and aged mice. The increased mortality of the aged mice during the 13-week DA exposure supports increased sensitivity to DA exposure with age. Poorer health outcomes with age following DA exposure are also supported by our FI data. Although the effect of the DA treatment on FI was not different between young adult and old mice (Figure 2c), the aged mice started at a lower health status, so the added effect of DA resulted in worse outcomes (i.e., greater frailty) in the aged mice (Figure 2b). The greater mortality and worse health outcomes are consistent with our previous results showing that aged mice were more sensitive to acute DA exposure [26]. This previous study also reported higher levels of DA in both tissue and blood with age following acute exposure, suggesting impaired clearance of DA through the kidney. Compromised renal clearance with age was previously suggested as a potential factor for the more severe DA impacts observed in older people during the first recognized human DA poisoning event [4,5]. Additional laboratory studies have documented the rapid renal clearance of DA in healthy young adult mammals [27,28]. Therefore, the greater mortality in this study is likely due to a combination of reduced resilience with age and greater physiological exposure to DA in the aged mice associated with poorer renal clearance [26].

Despite early clinician reports of arrhythmia in humans suffering DA poisoning, as well as comprehensive reviews of multiple potential toxicologic pathology targets [4,29], the majority of laboratory studies have focused on neurological endpoints. DA is a well-described neurotoxin with demonstrated effects on learning and memory with repeated low-level and acute exposures [20,30]. However, several lines of evidence suggest DA has significant cardiotoxicity [29]. Here, we report cardiomyopathy associated with DA exposures in a laboratory animal model with controlled, repeated, low-level exposure in as little as three weeks. Longer-term exposure to DA also led to diastolic dysfunction that phenocopied heart failure with preserved ejection fraction (HFpEF), which is associated with both mouse and human cardiac aging. In contrast to previous reports that the learning and memory deficits induced by low-level DA exposure in mice are reversible after a prolonged period of no exposure [20], the cardiac defects observed here after 13 weeks of exposure and ten weeks of recovery (no exposure) were not reversible, suggesting that this exposure led to permanent cardiac pathology.

Despite the greater mortality (Table 1) and higher FI score (Figure 2) of aged mice in the 13-week exposure condition (Table 1) and the higher FI score in the three-week exposure (Figure 2), the cardiac function of the aged mice in the three-week study was not impacted more by DA than that of the young adult mice (Figure 3). In the shorter study, we observed both diastolic (E’/A’) and systolic (FS) cardiac defects in young adult mice with DA treatment (Figure 3a,b). It is not clear why DA altered systolic function after only three weeks. It should be noted that FS, which was the only systolic measure assessed in the 13-week study, was not different after 13 weeks (Figure 1e), and the aged mice in the three-week study appeared healthier than those used in the 13-week study, as is clear from the absence of age-related deficits in cardiac function at baseline between young adult and aged mice. Other possible differences may be due to improved, more sensitive echocardiography in the second study, due to an improved system, and the implementation of global longitudinal strain analysis, which is regarded as the more sensitive measure of mouse systolic cardiac function [31].

Despite the primary focus on neurotoxicity, previous reports from environmentally exposed marine mammals corroborate our observations of cardiac pathologies from DA. Necropsies of California sea lions (CSLs; *Zalophus californianus*) with histories of exposure have identified a DA-associated, potentially lethal degenerative cardiomyopathy [31], and cardiac lesions, myofiber necrosis, edema, and nuclear abnormalities are regularly reported in CSL and northern fur seal (NFS; *Callorhinus ursinus*) heart tissue following DA exposure [22,32,33]. Furthermore, a 2005 study of southern sea otters (*Enhydra lutris nereis*) calculated an odds ratio of 10.6 for dilated cardiomyopathy (DCM) following suspected DA exposure [34]. A recent longitudinal study of 186 free-ranging sea otters from 2001 to 2017 used Bayesian spatiotemporal models to approximate DA exposure and found a strong association between chronic environmental DA exposure and fatal cardiac disease [35]. Interestingly, in that study, DA exposure was found to be associated with a greater risk for cardiomyopathy in prime-age adults, as opposed to otters of more advanced age, consistent with our observation that aged hearts are not more susceptible to DA-induced cardiac dysfunction.

This greater effect of DA on young adult hearts extended to analyses of the cardiac proteome. Over 70% of the proteins differentially expressed with age alone were also differentially expressed by three weeks of DA exposure in the young adult mice, while only 3% were altered by DA in the aged mice. One interpretation of this striking interaction between DA and age on the cardiac proteome is that DA induces an aging-like shift in the cardiac phenotype, at both the functional and proteomic levels. Therefore, in the aged mice, the cardiac proteomes are already remodeled, and subsequent DA exposure has more subtle effects that were not detectable at the protein expression level. Given the large overlap in individual protein expression, it is not surprising that pathway analyses yielded a similar pattern, where there were 15 pathways that were shared between aging and young DA exposure. Interestingly, most of the significantly altered pathways were downregulated by DA (Table 2). Protein components of the electron transport system, especially complex I, were highly represented in the downregulated pathways. Two pathways with increased expression with older age and DA treatment in the young mice involved ribosome and protein translation. Given the role of calcium in mitochondrial toxicity and the excitotoxic mechanism of DA that leads to disruption of calcium homeostasis, it is not surprising that mitochondria are a target of DA in the heart. The downregulation of mitochondrial electron transport system proteins is consistent with our previous report of mitochondrial dysfunction and subsequent oxidative and inflammatory stress following low-level DA exposure in zebrafish brain [36]. It is also noteworthy that despite the decline in electron transport system proteins, proteins involved in 2-oxocarboxylic acid metabolism were upregulated with DA treatment in young mice and in saline-treated old control mice (Table 2). This category consists of several TCA cycle and intermediary mitochondrial proteins involved in bioenergetic and amino acid biosynthetic reactions associated with age-related cardiac dysfunction.

While consistent with the known mechanism of DA toxicity, this report focuses on the functional cardiac effects and does not explicitly address potential mechanisms of DA cardiotoxicity. Though more research is needed, the prevailing hypothesis is that in cases where cardiac dysfunction contributes to morbidity or mortality, toxicity results from conduction disturbances and apoptotic pathway activation following DA’s direct interaction with cardiac glutamate receptors, not a centrally mediated brain-heart etiology [22]. This report and the present study clearly demonstrate that moving forward, DA research should continue to include assessments of peripheral organ system effects, in addition to neurological ones, as health effects from chronic low-level DA exposure may manifest through more pathways than those traditionally associated with acute exposure.

## 4. Materials and Methods

All animal experiments were approved by the University of Washington Institutional Animal Care and Use Committee.

### 4.1. Experiment 1: Long-Term Low-Level DA Exposure (13 Weeks)

Young adult and aged female C57Bl/6 mice (9 and 24 months (mo) old, respectively) were administered intraperitoneal injections (IP) of saline or 0.5 mg/kg bw DA in saline three times per week for a total of 13 weeks (Figure 1). This dose was chosen based on previous studies defining sub-convulsive DA exposure doses in mice [20]. After 13 weeks of exposure, young adult and aged mice underwent echocardiography to measure cardiac function. Because mortality in the aged mice was higher than expected during both the exposure period and the echocardiography procedure, aged mice were euthanized by cervical dislocation at this point. Young adult mice, all of whom survived the first echocardiography, were then allowed a recovery period of 10 weeks, during which no DA or saline was administered. At the end of that period, echocardiography was performed again.

### 4.2. Experiment 2: Short-Term Low-Level DA Exposure (Three Weeks)

Because of the unexpected deaths of the aged mice in the 13-week DA exposure treatment group, a second experiment with a shorter exposure period was performed to test the effect of age on low-level DA exposure (n = 10 per treatment; Figure 2a). Adult (9 mo) and aged (24 mo) female C57Bl/6 mice were administered IP-injected saline or a sub-convulsive dose of 0.5 mg/kg body weight DA in saline three times per week for a total of three weeks [20]. After two weeks of exposure, young adult and aged mice underwent echocardiography to measure cardiac function, and then continued with IP DA or saline injections for another week. By the end of the third week, FI assessments and echocardiography to measure cardiac function were performed. At the conclusion of these tests, all mice were also euthanized by cervical dislocation, and their hearts were frozen for protein analyses.

### 4.3. Frailty Index Assessment

A 29-item Frailty Index (FI) assay was performed on all mice in Experiment 2 before and after three weeks of DA or saline exposure. The FI assay was performed as described in Whitehead et al. 2014 to provide a rapid, non-invasive assessment of general health status [37]. FI was performed by the same researcher for all animals pre- and post-exposure. Each variable was given a score of 0, 0.5, or 1.0 if there was no deficit, mild deficit, or severe deficit, respectively. The FI score is calculated by adding the total points and dividing by the number of variables.

### 4.4. Echocardiography Imaging and Analysis for Cardiovascular Function

Echocardiography was performed using the Vevo 3100 preclinical imaging system, Vevo Imaging Station, and MS400 probe from VisualSonics (Toronto, ON, Canada). The mice were placed in a supine position, and HR, respiration, and core body temperature were monitored using the Vevo Animal Monitoring system SM200 from VisualSonics. The SM200 also provides heat support to the mice while they are maintained on 1–3% isoflurane anesthesia. Parasternal short-axis view (PSAX) B-mode and M-Mode images were acquired. Echocardiography was analyzed using the Vevo LAB software 5.6.0. ECG and heart rate were monitored throughout the procedure, with the mice’s heart rates being maintained in the range of 400–500 bpm at low workload. Systolic functions, including LV mass, ejection fraction, and fractional shortening, were quantified. Strain analysis (short-term DA exposure only), including global longitudinal strain (GLS), was analyzed from 5 consecutive cardiac cycles without respiration from the PLAX B-mode images using the Vevo Strain package. Diastolic function was assessed by tissue velocity from a tissue Doppler apical four-chamber view. In brief, an average of at least 3 individual cardiac cycles without respiration was used to calculate peak early (E’) and peak late (A’) mitral valve annulus tissue velocity. The results were exported to Microsoft Excel, and statistical analysis and graphing were performed using GraphPad Prism9 software.

### 4.5. Proteomics Methods

Proteomics data were filtered to remove any protein with >20% missingness, median normalized, and then missing data were imputed using K-nearest neighbors with 5 neighbors. The Bioconductor limma package [38] was used to make comparisons between groups, using the limma-trend pipeline [39] to account for heteroscedasticity, and sample weights to reduce the impact of possible outlier samples. All samples were included in the regression model, and individual comparisons were made using empirical Bayes-adjusted contrasts. Differentially abundant proteins were identified using a false discovery rate (FDR) <0.05. Advaita iPathwayGuide was used to identify differentially affected pathways (Advaita Corporation, Ann Arbor, MI, USA).

Tissue preparation: The tissues were chopped and extracted with 1% sodium deoxycholate in 100 mM ammonium bicarbonate by ultrasonication on PIXUL HT (Matchstick, Kirkland, WA, USA). Extracted samples were spun at 21k rpm for 30 min at 4 °C, and protein concentration was determined using the BCA assay. An aliquot of 10 μg of the total protein was then diluted to 0.1% SDS/100 mM ammonium bicarbonate, subjected to reduction and alkylation with dithiotreitol and iodoacetamide, and digested with trypsin (1 μg) overnight at 37 °C. Digestion was quenched, and SDC precipitated with formic acid (1% final concentration); the supernatant was then collected, cleaned using an Oasis HLB uElute plate, dried down, and stored at −20 °C until LCMS analysis. The samples were reconstituted in 1% acetonitrile/0.1% formic acid at 75 ng/μL.

LC-MS/MS analysis: Proteins in samples (n = 5 per treatment group) were identified and quantified by mass spectrometry using data-independent analysis (DIA) LC-MS/MS [40]. The 450 ng of the digested proteins were injected on a C18 trapping column (Reprosil-Pur 120 C18-AQ, 5 µm, 0.1 × 40 mm, Dr. Maisch HPLC GmbH, Ammerbuch, Germany) (flow rate 4 mL/min), and separated on an analytical column (Reprosil-Pur 120 C18-AQ, 5 µm, 250 × 0.075 mm, Dr. Maisch HPLC GmbH, Ammerbuch, Germany), with the following multi-step linear gradient: 1–7%B in 7 min, 7–28% in 105 min, 28–44% in 15 min, 44–100% in 4 min. They were then held for another 5 min, followed by reequilibration (A—0.1% formic acid in water, B—acetonitrile, 0.1% formic acid, flow rate of 0.4 µL/min). An LC-MSMS consisting of a Thermo EasyLC 1200 and a Thermo Orbitrap Exploris480 (Thermo Fisher, San Jose, CA, USA) mass spectrometer with electrospray ionization was used for the analysis.

Data-independent analysis parameters were as follows: MS1 scan (430–670 Da, resolution 120,000, maximum injection time 50 ms) followed by MS/MS scans across a 430–670 Da range with a 4 Da mass selection window each (resolution 30,000, maximum injection time 10 ms). Fragmentation was induced by HCD activation at a normalized collision energy level of 28%.

Peptide and protein detection and quantification were accomplished in a two-step process, with identification using FragPipe 4.4.1 and MSFragger 4.4.1 [41] to generate a peptide spectral library with fully tryptic peptides allowing for one missed cleavage, alkylated Cys residue, and variable oxidized Met and N-terminal acetylation residues, precursors, and a fragment mass tolerance of 20 ppm (Uniprot mouse reference proteome, Uniprot Release 2022_05). The peptide library was subsequently used in DIA-NN 1.8.2 beta 27 [42] processing to quantify peptides and proteins with parameters matching the MSFragger search parameters.

## 5. Conclusions

To our knowledge, this is the first study to assess cardiac function in vivo in a controlled laboratory study using low-level sub-convulsive DA exposure doses in a mammalian model. The results confirm that long (13 weeks)- and short (three weeks)-term low-level DA exposure causes irreversible cardiomyopathy and protein remodeling that appears to phenocopy aspects of natural cardiac aging. In short, it makes young hearts function like old hearts. Future work should also consider investigating overt structural damage or fibrosis in the heart with low-level exposure, as this could explain the persistence of observed cardiac dysfunction after a long recovery period. These findings have profound implications for human health if the observed cardiac dysfunction and aging phenotype also occur in human seafood consumers known to regularly consume low levels of DA [24].

## Figures and Tables

**Table 1:** Survivorship in young adult (9 mo at start of experiment) and old (24 mo at start of experiment) female C57Bl/6 mice administered saline (S) or 0.5 mg/kg bw domoic acid (DA) for a 13-week exposure period, echocardiography-associated anesthesia, and then a ten-week recovery period with no exposure. N/A = recovery echocardiography was not performed on old mice due to low survivorship.

Survivorship (%)				
Treatment	Starting Sample Size	13 Weeks of Exposure	13–Week Echocardiography	10–Week Recovery Period
Young-S	13	92	92	92
Young-DA	12	100	100	100
Old-S	11	82	73	N/A
Old-DA	13	31	23	N/A

**Table 2:** Significantly differentially expressed pathway comparisons (FDR < 0.05) between young adult domoic acid exposed (Young-DA) and young adult saline (Young-S); old domoic acid (Old-DA) exposed and old saline (Old-S); and Old-S and Young-S control mice. Bold font = differentially expressed pathways shared between DA exposure in young adult mice and natural aging. UP = upregulated pathway. DOWN = down-regulated pathway. ND = no difference. MIX = both up- and downregulation.

Pathway	Young-DA vs. Young-S	Old-DA vs. Old-S	Old-S vs. Young-S
Ribosome	**UP**	ND	**UP**
Thermogenesis	**DOWN**	ND	**DOWN**
Non-alcoholic fatty liver disease	**DOWN**	ND	**DOWN**
Parkinson disease	**DOWN**	ND	**DOWN**
Oxidative Phosphorylation/Mitochondria	**DOWN**	ND	**DOWN**
Huntington disease	**DOWN**	ND	**DOWN**
Prion Disease	**DOWN**	ND	**DOWN**
Diabetic cardiomyopathy	**DOWN**	ND	**DOWN**
COVID-19	**UP**	ND	**UP**
Amyotrophic lateral sclerosis	**DOWN**	ND	**DOWN**
Retrograde endocannabinoid	**DOWN**	ND	**DOWN**
Alzheimer’s disease	**DOWN**	ND	**DOWN**
Valine, leucine, isoleucine degradation	UP	ND	ND
2-oxocarbolic acid	**UP**	ND	**UP**
Chemical carcinogenesis	**DOWN**	ND	**DOWN**
Platelet activation	**DOWN**	ND	**DOWN**
Pathways of neurodegeneration	ND	ND	DOWN
Metabolic Pathways	ND	ND	MIX
Lipoic acid metabolism	ND	ND	UP
Propanoate metabolism	ND	ND	UP
Aminoacyl-tRNA biosynthesis	ND	ND	UP
Carbon metabolism	ND	ND	UP
Citrate cycle (TCA cycle)		ND	UP

## Data Availability

The original contributions presented in this study are included in the article. Further inquiries can be directed to the corresponding author.
